# Comparative analysis of the lung microbiota in patients with respiratory infections, tuberculosis, and lung cancer: A preliminary study

**DOI:** 10.3389/fcimb.2022.1024867

**Published:** 2022-11-01

**Authors:** Xiaoxue Xia, Jiang Chen, Yiwen Cheng, Feng Chen, Huoquan Lu, Jianfeng Liu, Ling Wang, Fengxia Pu, Ying Wang, Hua Liu, Daxing Cao, Zhengye Zhang, Zeping Xia, Meili Fan, Zongxin Ling, Longyou Zhao

**Affiliations:** ^1^ Department of Infectious Diseases, Changxing People’s Hospital, Huzhou, China; ^2^ Department of Neurosurgery, Changxing People’s Hospital, Huzhou, China; ^3^ Collaborative Innovation Center for Diagnosis and Treatment of Infectious Diseases, State Key Laboratory for Diagnosis and Treatment of Infectious Diseases, National Clinical Research Center for Infectious Diseases, The First Affiliated Hospital, School of Medicine, Zhejiang University, Hangzhou, China; ^4^ Jinan Microecological Biomedicine Shandong Laboratory, Jinan, China; ^5^ Department of Respiratory, Changxing People’s Hospital, Huzhou, China; ^6^ Department of Laboratory Medicine, Lishui Second People’s Hospital, Lishui, China

**Keywords:** community-acquired pneumonia, inflammation, lung cancer, lung microbiota, pulmonary tuberculosis

## Abstract

Recent evidence suggests that lung microbiota can be recognized as one of the ecological determinants of various respiratory diseases. However, alterations in the lung microbiota and associated lung immunity in these respiratory diseases remain unclear. To compare the lung microbiota and lung immune profiles in common respiratory diseases, a total of 78 patients were enrolled in the present study, including 21 patients with primary pulmonary tuberculosis (PTB), eight patients with newly diagnosed lung cancer (LC), and 49 patients with community-acquired pneumonia (CAP). Bronchoalveolar lavage fluid (BALF) was collected for microbiota and cytokine analyses. With MiSeq sequencing system, increased bacterial alpha-diversity and richness were observed in patients with LC than in those with PTB and CAP. Linear discriminant analysis effect size revealed that CAP-associated pulmonary microbiota were significantly different between the PTB and LC groups. More key functionally different genera were found in the PTB and LC groups than in the CAP group. The interaction network revealed stronger positive and negative correlations among these genera in the LC group than in the other two groups. However, increased BALF cytokine profiles were observed in the PTB group than in the other two groups, while BALF cytokines were correlated with key functional bacteria. This comparative study provides evidence for the associations among altered lung microbiota, BALF inflammation, and different respiratory disorders, which provides insight into the possible roles and mechanisms of pulmonary microbiota in the progression of respiratory disorders.

## Introduction

Trillions of microorganisms, such as bacteria, archaea, viruses, fungi, and protozoans, live in various human habitats, such as the gastrointestinal tract, respiratory tract, urogenital tract, oral cavity, and skin, which play essential roles in maintaining human health (Ling et al., 2022a; Li et al., 2022). Among all host microbial habitats, the gut microbiota has attracted the most attention in current biomedical research, whereas the study of the respiratory microbiota is a relatively new field (Man et al., 2017). Like the gastrointestinal tract, the respiratory tract is also a complex system that extends from the nasal cavity, nasopharynx, oropharynx, and trachea straight to the lungs, and is divided into the upper and lower respiratory tracts, which harbor complex, distinct, and specialized microbial communities (Marsland et al., 2013). In the upper and lower respiratory tracts, respiratory microbiota contain niche-specific symbiotic and pathogenic microorganisms, which play a significant role in determining the occurrence and development of diseases. Generally, the lung microbiota seem to be dominated by Firmicutes and Bacteroidetes members (Man et al., 2017), whereas the microbiota of the upper respiratory tract are enriched in Firmicutes and Actinobacteria members in the nostrils and Firmicutes, Proteobacteria, and Bacteroidetes members in the oropharynx (Lemon et al., 2010). Respiratory microbiota act as gatekeepers in maintaining human health, protecting the body from pathogens by colonizing mucosal surfaces and secreting various antimicrobial peptides, including short-chain fatty acids or host-derived cytokines and chemokines (Budden et al., 2019; Kim and Jang, 2020). In addition, the respiratory microbiota interact with the host immune system, affecting the clinical outcomes in chronic and acute respiratory diseases (Man et al., 2017). Alterations in respiratory microbiota are strongly associated with various respiratory disorders, such as asthma (Huang et al., 2015), allergies (Salameh et al., 2020), lower respiratory tract infections (Lanaspa et al., 2017; Qin et al., 2022), pulmonary tuberculosis (PTB) (Eshetie and Van Soolingen, 2019), and cystic fibrosis (Wypych et al., 2019). The altered microbial biodiversity and decrease in beneficial commensals in the respiratory microbiota may contribute to the pathogenesis of these respiratory diseases. Respiratory microbiota research can reveal more roles of the microbiota in human health and diseases and decipher the pathogenic mechanisms of various lung and airway conditions.

Lower respiratory tract disorders, such as pneumonia, tuberculosis, and lung cancer (LC), are the most common respiratory diseases and leading causes of morbidity and mortality worldwide (GBD 2016 Lower Respiratory Infections Collaborators, 2018; De Martel et al., 2020; Cao et al., 2021). To some extent, the occurrence and development of these lower respiratory tract disorders may be linked to the disruption in the stability of respiratory microbiota, as the pulmonary microbiota are key to maintaining stable respiratory immune homeostasis, maturation, and respiratory physiology (Man et al., 2017). Dysbiotic pulmonary microbiota can directly impact immunity or alter the local immunity/inflammation during the development of diseases, which may hypothetically be associated with clinical worsening of the disease due to immune dysregulation, excessive inflammation and/or infection (Xiao et al., 2022). However, alterations in the pulmonary microbiota and related systemic immune disturbances have not yet been extensively explored. It is yet to be definitively established whether specific members of the pulmonary microbiota provide direct stimuli that lead to disease susceptibility or protection. Thus, understanding this relationship is fundamental in the search for more effective therapies for respiratory diseases.

Compared to gut microbiota research, pulmonary microbiota research is a relatively new field that is impeded by the availability of sampling methods. Sampling of healthy subjects for research purposes is difficult due to its invasive nature, which is why healthy pulmonary microbiota are yet to be defined. Several sample types, including oropharyngeal swabs or washes, sputum samples, bronchial aspirates, bronchoalveolar lavage fluids (BALFs), and endobronchial biopsies have been extensively used to investigate the lung microbiota. Among these samples, BALF, which is obtained *via* bronchoscopy, enables the identification of microbiota specific to the lower respiratory tract, but commonly has low bacterial density (Schneeberger et al., 2019), generally 2–4 logs lower than that in the upper respiratory tract (Dickson et al., 2017). BALF is more objective and representative than oropharyngeal swabs or sputum in reflecting the microbial environment of the lungs (Mimoz and Dahyot-Fizelier, 2007; Jin et al., 2019). Thus, BALF is considered as the ideal carrier of representative information about lung microbiota and the optimal sample choice for lung microbiota analysis (Bingula et al., 2020). However, until recently, there have been only a few studies on the pulmonary microbiota in patients with lower respiratory tract disorders. The potential roles of pulmonary microbiota in these disorders are yet to be defined. In the present study, we sought to compare the bacterial microbiota and immune profiles of BALF samples from patients with community-acquired pneumonia (CAP; n=49), PTB (n=21), and LC (n=8) using 16S rRNA-based next-generation sequencing, which is important to understand the pathogenesis of these diseases and develop effective microbiota-based therapeutic interventions.

## Methods

### Participants’ enrollment

Application of the study was approved by the Ethics Committee of Changxing People’s Hospital (Huzhou, Zhejiang, China; Reference number: 2018-004). The study was conducted under the principles of the Helsinki Declaration and written informed consent was obtained from each participant prior to enrollment. 78 patients including 21 patients with PTB, eight patients with newly diagnosed LC, and 49 patients with CAP admitted to our hospital between February 2021 and December 2021 were enrolled. All these patients were diagnosed *via* case history, chest radiography, and blood markers examinations following Chinese clinical guidelines (Commission, N.H., 2020; He et al., 2021; Qu et al., 2022). We also collected the demographic data and medical history of all study participants using a set of questionnaires, such as age, gender, ethnicity, body mass index (BMI), smoking history, smoking amount, comorbidities, lifestyle, eating habits and so on. The exclusion criteria included: age < 18 or > 65 years; BMI  > 28 kg/m^2^; pregnant; atypical pneumonia; chronic obstructive pulmonary disease; allergenic diseases; diabetes mellitus; hypertension; gastritis; hepatitis; other cancers; antibiotics, prebiotics, probiotics, synbiotics, antitumor drugs, glucocorticoid or other immunosuppressants administration in the previous 1 month; other active and known infections.

### BALF sample collection and storage

All eligible study population underwent bronchoscopy, and BALF samples were collected from each participant using a standard clinical protocol by one expert. Bronchoscopy was performed within 48h after hospital admission. Prior to bronchoscopy, each patient signed the formal consent from before being undergone and understood the aims, methods, merits and risks of this operation in detail. Subjects received a topical anaesthesia (lidocaine) by nebulizer and then were sedated with midazolam and fentanyl. Bronchoscopy with BALF was performed according to standardized procedures designed to minimize oral contamination (Iwai et al., 2012). Specifically, bronchoalveolar lavage was performed by instilling 3 × 20 mL of sterile physiological saline into the bronchus. After each instillation, the maximum amount of liquid inside the bronchus was retrieved (nearly 10 mL in total) into 50 mL sterile plastic tube, and stored at -80°C after preparation within 15 min until use.

### Bacterial DNA extraction

The BALF samples underwent centrifugation at 10,000×g for 10 min and then precipitates and supernatants were separated for microbiota analysis and cytokines detection, respectively. The precipitate was re-suspended in 200 μL of phosphate buffered saline (PBS, GE HyClone). Bacterial genomic DNA was extracted using a QIAamp DNA Mini Kit (QIAGEN, Hilden, Germany) according to the manufacturer’s instructions. The amount of DNA was determined using a NanoDrop ND-1000 spectrophotometer (Thermo Electron Corporation, Boston, MA, USA) and the quality of DNA was checked by agarose gel electrophoresis. All DNA was stored at -20°C before further analysis.

### Amplicon library construction and sequencing

The protocols of amplicon library construction and sequencing were conducted as our previous studies (Ling et al., 2020a; Liu et al., 2021; Ling et al., 2022b). The details were shown as follows: amplicon libraries were constructed with Illumina sequencing-compatible and barcode-indexed bacterial PCR primers 341F (5’-CCTACGGGNGGCWGCAG-3’)/785R (5’-ACTACHVGGGTATCTAATCC-3’), which target the V3-V4 regions of the 16S rRNA gene. All PCR reactions were performed with KAPA HiFi HotStart ReadyMix using the manufacturer’s protocol (KAPA Biosystems) and approximately 50 ng of extracted DNA per reaction. Thermocycling conditions were set at 95°C for 1 min, 55°C for 1 min, then 72°C for 1 min for 30 cycles, followed by a final extension at 72°C for 5 min. All PCR reactions were performed in 50 μl triplicates and combined after PCR. The amplicon library was prepared using a TruSeq™ DNA sample preparation kit (Illumina Inc, San Diego, CA, USA). Prior to sequencing, the PCR products were extracted with the MiniElute^®^ Gel Extraction Kit (QIAGEN) and quantified on a NanoDrop ND-1000 spectrophotometer (Thermo Electron Corporation) and Qubit 2.0 Fluorometer (Invitrogen). The purified amplicons were then pooled in equimolar concentrations and the final concentration of the library was determined by Qubit (Invitrogen). Negative DNA extraction controls (lysis buffer and kit reagents only) were amplified and sequenced as contamination controls. Sequencing was performed on a MiSeq instrument (Illumina) using a 300 × 2 V3 kit together with PhiX Control V3. MiSeq sequencing and library construction were performed by technical staff at Hangzhou KaiTai Bio-lab.

### Bioinformatic analysis

According to our previous studies, the 16S rRNA gene sequence data set generated from the Illumina MiSeq platform was inputted to QIIME2 (version 2020.11), and all steps of sequence processing and quality control were performed in QIIME2 with default parameters (Ling et al., 2019; Liu et al., 2019; Ling et al., 2020a; Liu et al., 2021; Ling et al., 2022b). Before the following data analysis, these reads of each sample were normalized to even sampling depths and annotated using the Greengenes reference database (version 13.8) with both the RDP Classifier and UCLUST version 1.2.22 methods implemented in QIIME2. α-diversity indices, including the observed species, abundance-based coverage estimator (ACE), Chao1 estimator, Shannon, Simpson, Evenness and PD whole tree indices, were calculated at a 97% similarity level. β-diversity was measured by the unweighted UniFrac, weighted UniFrac, Jaccard and Bray-Curtis distances calculated by QIIME2, which were visualized by principal coordinate analysis (PCoA). The differences in the composition of the fecal microbiota at different taxonomic levels were analyzed with Statistical Analysis of Metagenomic Profiles (STAMP) software package v2.1.3 and the linear discriminant analysis (LDA) effect size (LEfSe) method. Krona chart was plotted using taxonomy summary data obtained from QIIME Krona chart displays abundance and hierarchy simultaneously using a radial space-filling display and features a red-green color gradient, signifying the average BLAST hits e-values within each taxon (Ondov et al., 2011). PiCRUSt v1.0.0 was used to identify predicted gene families and associated pathways from inferred metagenomes of taxa of interest identified from the compositional analyses.

### Multiplex cytokine analysis

According to our previous studies, BALF cytokines, chemokines and growth factors were probed using Bio-Plex Pro Human Cytokine 27-plex Panel (M50-0KCAF0Y, Bio-Rad, Hercules, CA, USA) multiplex magnetic bead-based antibody detection kits following the manufacturer’s instructions. Based on the Luminex^®^ xMAP^®^ technology, the assays are capable of simultaneously quantifying 27 targets including interleukin-1β (IL-1β), IL-1 receptor antagonist (IL-1ra), IL-2, IL-4, IL-5, IL-6, IL-7, IL-8, IL-9, IL-10, IL-12(p70), IL-13, IL-15, IL-17, Eotaxin, Fibroblast growth factor-basic (FGF-basic), granulocyte colony-stimulating factor (G-CSF), granulocyte-macrophages colony-stimulating factor (GM-CSF), interferon gamma (IFN-γ), interferon gamma-inducible protein 10 (IP-10), monocyte chemotactic protein-1 (MCP-1), macrophages inflammatory protein-1α (MIP-1α), platelet-derived growth factor (PDGF-bb), MIP-1β, regulated upon activation normal T-cell expressed and secreted (RANTES), tumor necrosis factor-alpha (TNF-α), and vascular endothelial growth factor (VEGF). The assays were run on the Luminex^®^ 200™ system (Bio-Rad) and fluorescence values were collected. A standard curve was derived using the different concentrations of the assay standards. Data was acquired using the Bio-Plex Array Reader system 2200. The results expressed as picogram per milliliter (pg/mL) using the standard curves integrated into the assay and Bio-Plex Manager v5.0 software with reproducible intra- and inter-assay CV values of 5-8% (Ling et al., 2020a; Ling et al., 2020b).

### Statistical analysis

White’s nonparametric *t*-test, independent *t*-test, or Mann-Whitney *U*-test were applied for continuous variables. Pearson chi-square or Fisher’s exact test were used for categorical variables between groups. Spearman’s rank correlation test was utilized for correlation analyses. Statistical analysis was performed using the SPSS v24 (SPSS Inc., Chicago, IL) and STAMP v2.1.3 (Parks et al., 2014). R packaged and GraphPad Prism v6.0 were used for preparation of graphs. All tests of significance were two sided, and p<0.05 or corrected p<0.05 was considered statistically significant.

### Accession number

The sequence data from this study are deposited in the GenBank Sequence Read Archive with the accession number PRJNA867528.

## Results

### Characteristics of participants

In the present study, 78 patients including 49 patients with CAP, 21 patients with primary PTB, and eight newly diagnosed patients with LC. No differences in clinical characteristics, such as age, sex, body mass index, smoking history, smoking amount, comorbidities, and eating habits, were detected among the three groups (p > 0.05). All participants were first examined, clinically diagnosed, and treatment-naïve. *Streptococcus pneumoniae* (n = 36) and *Haemophilus influenzae* (n = 13) were the two infectious microorganisms detected in patients with CAP. *Mycobacterium tuberculosis* isolated from patients with primary PTB was not a multidrug-resistant strain and could be treated with first-line anti-tuberculosis drugs. LC was pathologically confirmed as non-small cell LC.

### Comparison of pulmonary microbiota in patients with different respiratory disorders

**Figure 1:**
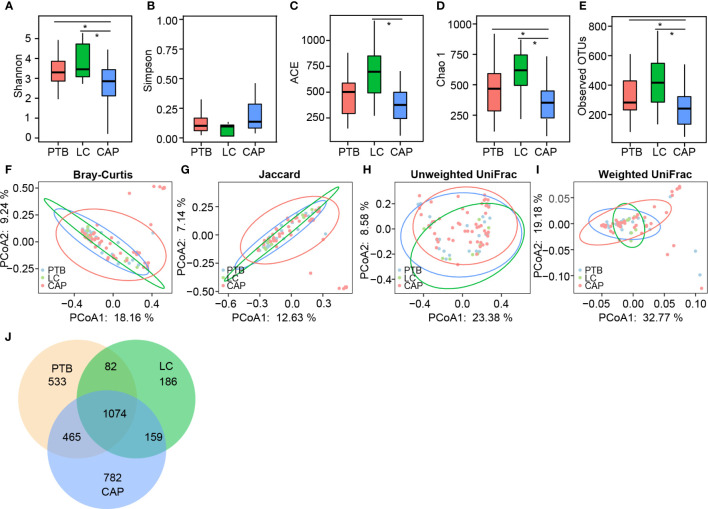
Comparison of the overall structure of lung microbiota in patients with primary pulmonary tuberculosis (PTB), newly diagnosed lung cancer (LC), and community-acquired pneumonia (CAP). The diversity indices of Shannon **(A)** and Simpson **(B)**, and the richness indices of ACE **(C)**, Chao1 **(D)**, and the observed OTUs **(E)** were used to evaluate the overall structure of the lung microbiota in different respiratory diseases. The data are presented as mean ± standard deviation. Unpaired *t* tests (two tailed) were used to analyze the variation between the groups. *p < 0.05 compared with the control group.. Principal coordinate analysis (PCoA) plots of individual lung microbiota based on Bray–Curtis **(F)**, jaccard **(G)**, and unweighted **(H)** and weighted **(I)** UniFrac distances in patients with different respiratory diseases. Each symbol represents a sample. The Venn diagram illustrates the overlap of OTUs in lung microbiota among different respiratory diseases **(J)**.

For microbiota analysis, we obtained 3,748,364 high-quality reads (1,017,729 for PTB group, 353,766 for LC group, and 2,376,869 for CAP group), with an average of 48,055 reads per sample. In total, we identified 3,281 operational taxonomic units (OTUs, unique bacterial phylotypes) among the pulmonary microbiota, attaining a Good’s coverage of 99.37%, indicating that most of the pulmonary bacteria were detected. For bacterial α-diversity analysis, a decreased Shannon index was found in patients with CAP than in those with PTB and LC (**Figure 1A**; p < 0.05), whereas the Simpson index was not significantly different among the three groups (**Figure 1B**). The richness index, such as ACE, was significantly higher in LC group than in CAP group (**Figure 1C**; p < 0.05), whereas the other two richness indices, such as Chao 1 and observed species, were significantly lower in CAP group than in the other two groups (**Figures 1D, E**; p < 0.05). For β-diversity analysis, PCoA plots were generated based on the Bray–Curtis, Jaccard, unweighted UniFrac, and weighted UniFrac distances to characterize the global differences among the pulmonary microbiota of the three groups (**Figures 1F–I**). Our results showed no significant differences among the different respiratory disorders (ADONIS test, p > 0.05). Additionally, according to the Venn diagram showing the shared OTUs among different respiratory disorders, there were a total of 1,074 shared OTUs among the three groups, while 533, 186, and 782 OTUs were unique to patients with PTB, LC, and CAP, respectively (**Figure 1J**). Our present data suggest that altered bacterial α-diversity in the pulmonary microbiota is detected in different respiratory disorders.

**Figure 2:**
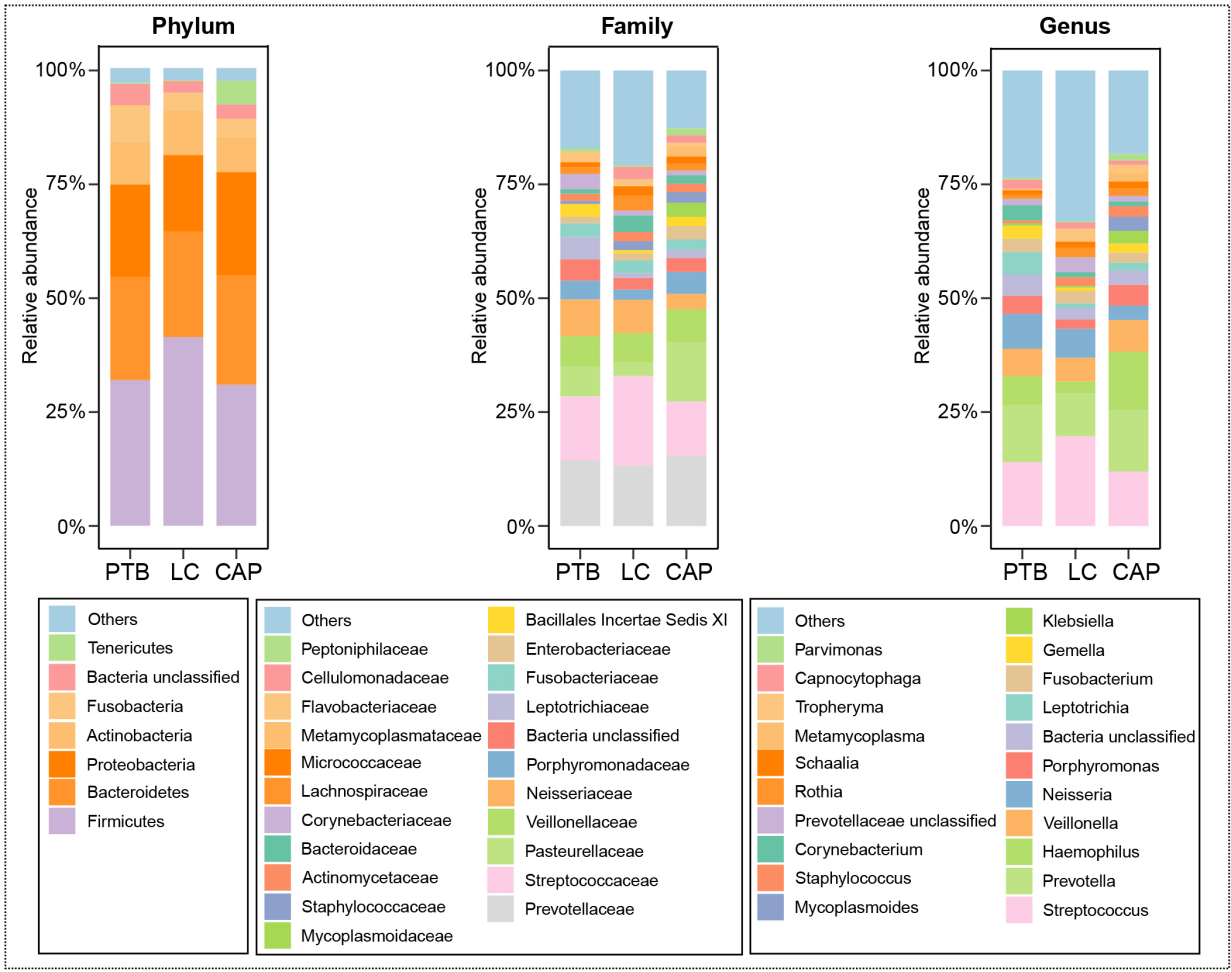
Relative proportions of bacterial phyla, families, and genera in lung microbiota in patients with primary pulmonary tuberculosis (PTB), newly diagnosed lung cancer (LC), and community-acquired pneumonia (CAP).

**Figure 3:**
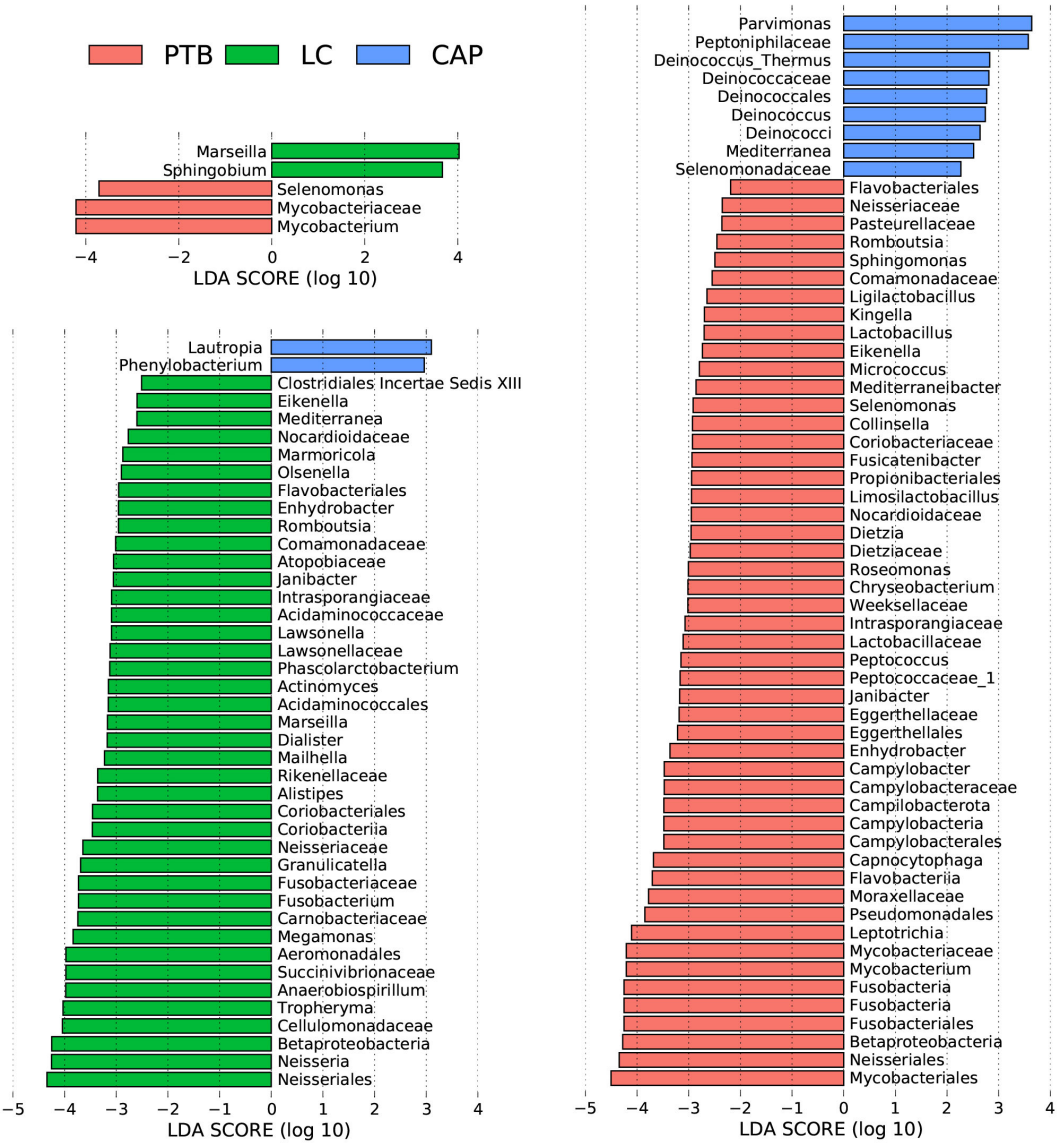
Taxonomic differences of the lung microbiota among patients with primary pulmonary tuberculosis (PTB), newly diagnosed lung cancer (LC), and community-acquired pneumonia (CAP). LEfSe identified the most differentially abundant taxa between the two groups. Only the taxa meeting a significant LDA threshold value of > 2 are shown.

For taxonomic analysis of pulmonary microbiota, we characterized the composition of bacterial communities at different taxonomic levels. In total, the sequencing reads were classified into 31 phyla, 223 families, and 509 genera in the pulmonary microbiota using the RDP classifier. **Figure 2** displays only the top seven phyla, top 21 families, and top 21 genera of the pulmonary microbiota in patients with different respiratory disorders. Krona radial space-filling charts showed the mean relative abundances of bacterial taxa in patients with different respiratory disorders from the phylum to genus level (starting at the inner circle) (**Figure S1**). These charts clearly demonstrate that the pulmonary microbiota were dominated by the phyla Firmicutes, Bacteroidetes, Proteobacteria, Actinobacteria, and Fusobacteria. LEfSe identified the differential bacteria at different taxonomic levels among the three groups (LDA score > 2.0, p < 0.05; **Figures 3**, **S2**). The composition of the pulmonary microbiota in the PTB and LC groups was similar, as only two genera, *Mycobacterium* and *Selenomonas*, were enriched in the PTB group, and the other two genera, *Sphingobium* and *Marseilla*, increased in the LC group. However, the pulmonary microbiota profiles of PTB and LC groups were distinct from those of the CAP group. Interestingly, 24 differential genera were observed between the LC and CAP groups, and only *Phenylobacterium* and *Lautropia* were enriched in the CAP group, while the other 22 genera, including *Neisseria*, *Megamonas*, and *Fusobacterium*, were significantly enriched in the LC group. When comparing the pulmonary microbiota between PTB and CAP groups, three genera, *Parvimonas*, *Deinococcus_Thermus*, and *Deinococcus*, were enriched in the CAP group, whereas 21 genera, including *Mycobacterium*, *Leptotrichia*, *Campylobacter*, and *Lactobacillus*, increased significantly in the PTB group. As identified by LEfSe, these differential genera can be used as candidate biomarkers to discriminate between different respiratory disorders.

**Figure 4:**
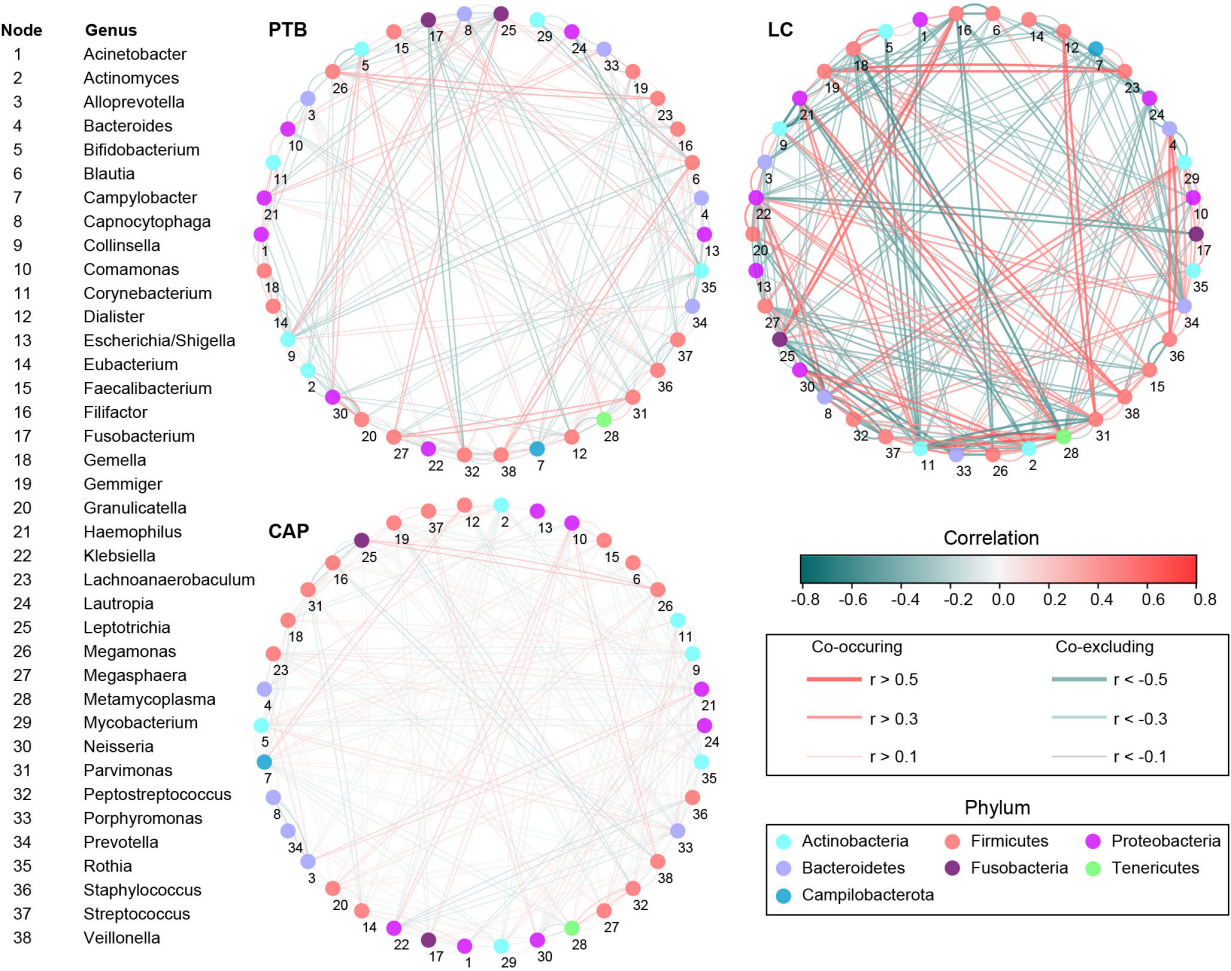
Correlation strengths of the abundant lung microbiota in patients with primary pulmonary tuberculosis (PTB), newly diagnosed lung cancer (LC), and community-acquired pneumonia (CAP). The correlation coefficients were calculated using the Sparse Correlations for Compositional data (SparCC) algorithm. Cytoscape version 3.4.0 was used for network construction. The red and blue lines represent positive and negative correlations, respectively. The correlation networks in LC were observed to become stronger and more complicated.

Additionally, the overall structure of the pulmonary microbiota was determined by the dynamic interactions between community members. Our SparCC algorithm with FDR adjustments was used to generate correlation-based networks of microbial interactions based on the relative abundance of OTUs between groups (**Figure 4**). Our present interaction network consisted of 38 nodes at the genus level (the most abundant bacterial genera), and stronger positive and negative correlations among the bacteria were found in the LC group than in the other two groups, suggesting that different respiratory disorders significantly influenced the interactions of the bacteria in the pulmonary microbiota. Taken together, the differences in the overall structure and composition of the pulmonary microbiota were evident among these respiratory disorders.

### Comparison of the general function profiles of the pulmonary microbiota among different respiratory disorders

**Figure 5:**
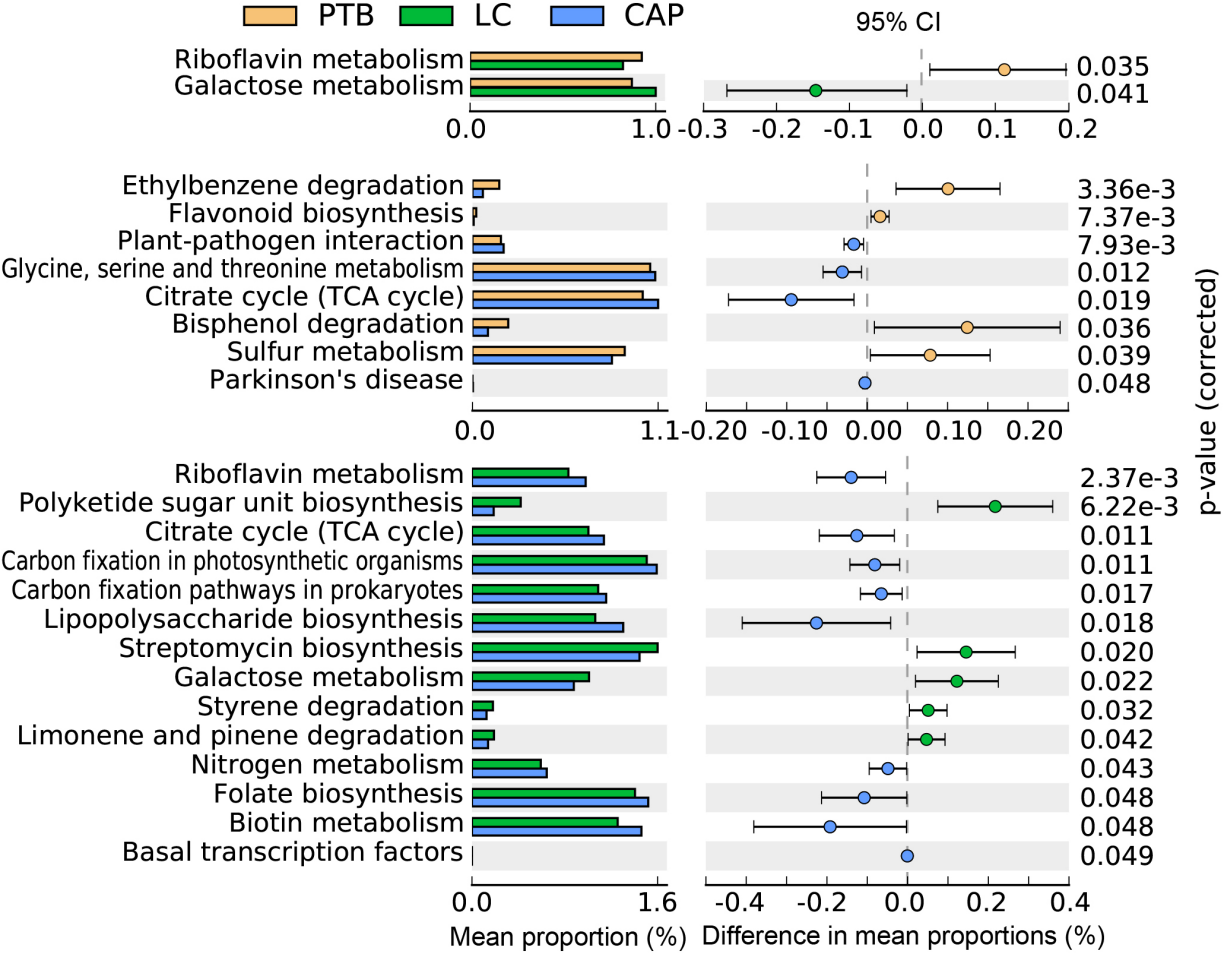
PiCRUSt-based examination of the lung microbiota of patients with primary pulmonary tuberculosis (PTB), newly diagnosed lung cancer (LC), and community-acquired pneumonia (CAP). The different bacterial functions were evaluated between them based on two-sided Welch’s *t*-test. Comparisons between the groups for each KEGG functional category (levels 3) are shown by percentage. The Benjamini–Hochberg method was used for multiple testing correction based on the false discovery rate (FDR) through STAMP.

In the present study, the bacterial functions of the pulmonary microbiota were explored using the PiCRUSt algorithm, which can predict the abundance of functional categories of the Kyoto Encyclopedia of Genes and Genomes (KEGG) ortholog (KO) based on closed-reference OTUs picking to identify metabolic and functional changes in the pulmonary microbiota. Comparisons of the general functional profiles of the pulmonary microbiota among the different respiratory disorders are shown in **Figure 5**. Similar to the changing patterns of pulmonary microbiota profiles, the predicted functions among different respiratory disorders were distinct. Specifically, only two categories, such as riboflavin metabolism and galactose metabolism, showed clear differential abundances between the patients with PTB and LC in KEGG level 3 (p < 0.05). However, more KEGG categories in the pulmonary microbiota were found to be different between the CAP group and the other two groups. Among these altered predicted functional categories, glycine, serine and threonine metabolism, and the citrate cycle decreased significantly in the PTB group, while sulfur metabolism, bisphenol degradation, ethylbenzene degradation, and flavonoid biosynthesis were enriched compared with the CAP group. Additionally, five pathways, streptomycin biosynthesis, polyketide sugar unit biosynthesis, galactose metabolism, styrene degradation, and limonene and pinene degradation pathways, showed higher activity, while nine other pathways, including lipopolysaccharide biosynthesis, biotin metabolism, folate biosynthesis, and nitrogen metabolism pathways, showed prominently decreased activity in LC-associated pulmonary microbiota compared with the CAP group. Collectively, the distinct functional potential of the bacterial assemblages in the pulmonary microbiota may actively participate in the pathogenesis and development of these respiratory disorders.

### Correlations between pulmonary genera and host cytokines

**Figure 6:**
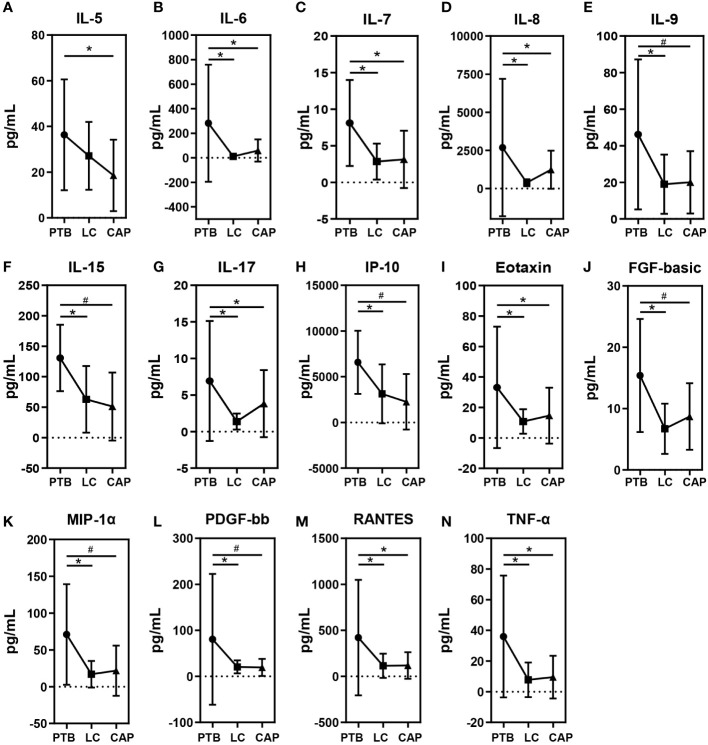
Mean ( ± SEM) concentrations (pg/ml) of 27 bronchoalveolar lavage fluids (BALF) pro- and anti-inflammatory cytokines and chemokines in patients with primary pulmonary tuberculosis (PTB), newly diagnosed lung cancer (LC), and community-acquired pneumonia (CAP). 14 BALF cytokines or chemokines such as IL-5 **(A)**, IL-6 **(B)**, IL-7 **(C)**, IL-8 **(D)**, IL-9 **(E)**, IL-15 **(F)**, IL-17 **(G)**, IP-10 **(H)**, Eotaxin **(I)**, FGF-basic **(J)**, MIP-1α **(K)**, PDGF-bb **(L)**, RANTES **(M)** and TNF-α **(N)** were found to be different among the three respiratory disorders. *p < 0.05 and #p < 0.01.

As shown in **Figure 6**, different respiratory disorders showed altered cytokine profiles in BALF. Of the 27 cytokine levels examined in the present study, 14 BALF cytokines or chemokines, including IL-5, IL-6, IL-7, IL-8, IL-9, IL-15, IL-17, IP-10, Eotaxin, FGF-basic, MIP-1α, PDGF-bb, RANTES, and TNF-α, were found to be different among the three respiratory disorders, while the other 13 cytokines were not obviously different. Interestingly, the changing patterns of BALF cytokines were almost similar between the LC and CAP groups (*: p < 0.05; #: p < 0.01 for each), which were significantly lower than those in the PTB group, while there were no significant differences between the LC and CAP groups (p > 0.05). Alterations in BALF cytokine profiles among different respiratory disorders were inconsistent with the changing patterns of the pulmonary microbiota.

**Figure 7:**
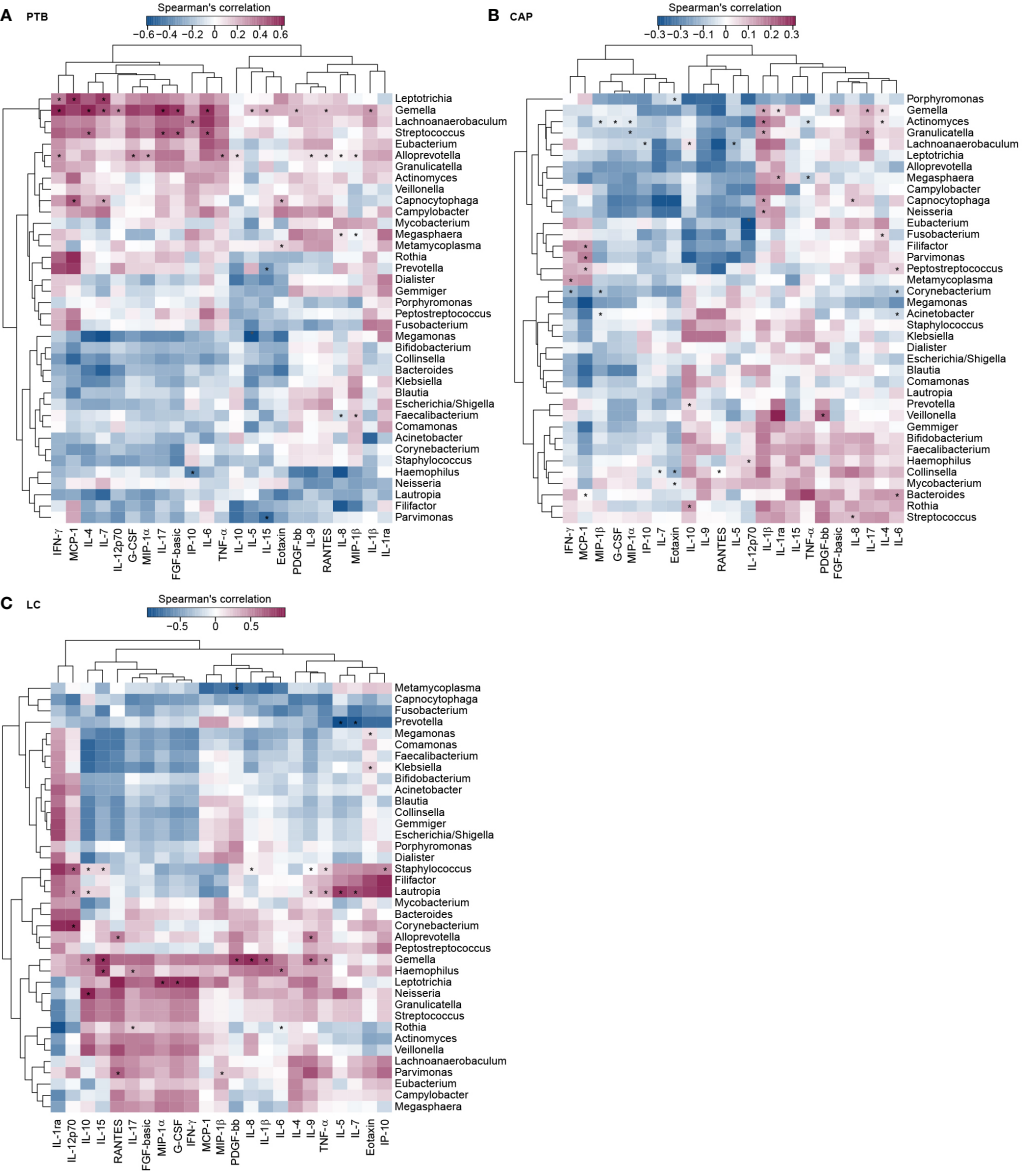
Correlations between lung microbiota, and bronchoalveolar lavage fluids (BALF) pro- and anti-inflammatory cytokines and chemokines in PTB **(A)**, CAP **(B)** and LC **(C)** patients. The heatmap shown Spearman’s correlation coefficients between differential genera and BALF cytokines. Spearman correlation (r) and probability (p) were used to evaluate statistical importance. *p < 0.01.

To assess the reciprocal relationship between altered BALF cytokines and key functional bacteria in the pulmonary microbiota from different respiratory disorders, we performed the correlation analyses using Spearman’s correlation separately. Heatmaps were created based on the Spearman’s correlation coefficients (r; p < 0.01) (**Figure 7**). In PTB patients, we found that *Leptotrichia*, and *Gemella* were positively correlated with IFN-γ, and IL-7; *Leptotrichia*, and *Capnocytophaga* were positively correlated with MCP-1, and IL-7; *Gemella* and *Streptococcus* were positively correlated with IL-4, and IL-17; whereas *Haemophilus* was negatively correlated with IP-10; *Prevotella* and *Parvimonas* were negatively correlated with IL-15. In CAP patients, we observed that *Streptococcus* and *Capnocytophaga* were positively correlated with IL-8; *Gemella* and *Capnocytophaga* were positively correlated with IL-1β; *Filifactor*, *Parvimonas*, and *Peptostreptococcus* were positively correlated with MCP-1; *Lachnoanaerobaculum*, *Prevotella*, and *Rothia* were positively correlated with IL-10; whereas *Corynebacterium* and *Granulicatella* were negatively correlated with MIP-1β; *Corynebacterium* was negatively correlated with IFN-γ, and IL-6. In LC patients, our correlation analysis demonstrated that *Staphylococcus*, *Lautropia* and *Gemella* were positively correlated with TNF-α; *Leptotrichia* was positively correlated with MIP-1α; *Parvimonas* was positively correlated with MIP-1β and RANTES; whereas *Prevotella* was negatively correlated with IL-5 and IL-7. The different correlation patterns suggested that the key functional lung bacteria played quite different roles in the development of different respiratory disorders.

## Discussion

Recent advances in culture-independent techniques have revealed complex microbial communities inhabiting healthy lungs, which has broken the long-held view that the lungs are sterile. With logarithmic improvements in sequencing technology and bioinformatics analysis, the study of lung microbiota has received increasing interest. In contrast to gut microbiota studies (Khan et al., 2022), the low concentration of microorganisms in the lung (10^3^ to 10^5^ bacteria per gram of tissue sample) and physiological and anatomic barriers leading to sampling, isolation, and estimation difficulties have delayed the understanding of the lung microbiota. The recent identification of the lung microbiota using high-throughput sequencing techniques has significantly revolutionized in this field. Currently, lung microbiota are recognized as one of the critical ecological determinants of lung wellness. In addition to symbiotic relationships, an emerging function of the lung microbiota is to promote and maintain immune homeostasis to prevent uncontrolled and undesirable inflammatory responses (Dickson and Huffnagle, 2015). Alterations in lung microbiota have been observed in many acute and chronic respiratory diseases, leading to disrupted homeostasis between the host and lung microbiota (Dickson et al., 2014; Taylor et al., 2016). These studies have highlighted the role of an imbalanced lung microbiota in the pathogenesis of respiratory diseases (Xu et al., 2020). Understanding the roles of lung microbiota in respiratory diseases may help to identify novel therapeutic targets and design new personalized therapeutic approaches to treat respiratory diseases.

We compared the overall structure and composition of the pulmonary microbiota among patients with PTB, LC, and CAP using high-throughput MiSeq sequencing technique for the first time in this study. BALF has been successfully used in various lung microbiota studies (Carney et al., 2020). In BALF samples, we observed increased α-bacterial diversity and richness in LC group compared with the other two groups, while the β-bacterial diversity was not significantly different among the three groups. Firmicutes, Bacteroidetes, Proteobacteria, Actinobacteria, and Fusobacteria were the dominant phyla in the pulmonary microbiota. The pulmonary microbiota in the CAP group was significantly different from those in the PTB and LC groups, whereas the pulmonary microbiota in PTB group was similar to that in the LC group. Several key functional differential genera were identified using LEfSe, which could be used as potential biomarkers to discriminate between different respiratory disorders. We also found stronger positive and negative correlations between these bacteria in the LC group than in the other two groups. Additionally, different bacterial functions of the pulmonary microbiota were observed among different respiratory disorders. We also found that different respiratory disorders showed altered BALF cytokine profiles, which correlated with key functional bacteria in the pulmonary microbiota. These differences might be due to the markedly different environmental conditions on the lung epithelial surfaces in different respiratory disorders, resulting in markedly different microbial communities and immune system interactions.

Using culture-independent techniques, previous human and animal studies have found that healthy lungs harbor a small community of bacteria, mainly comprising four phyla: Firmicutes, Proteobacteria, Bacteroidetes, and Actinobacteria (Morris et al., 2013). It has been proposed that microaspiration of pharyngeal secretions in healthy subjects is the main source of lung microbiota (Gopalakrishnan et al., 2018). Various oral commensals, such as *Prevotella*, *Veillonella*, and *Streptococcus*, are found in the lungs of most healthy individuals (Tsay et al., 2018; Wypych et al., 2019). While being a small amount but important, like the functions of gut microbiota in shaping immune system development in the gastrointestinal tract, pulmonary microbiota have been gradually realized to play important roles in maintaining homeostasis in the lung (Yang et al., 2020). Almost all types of pulmonary diseases, such as LC, chronic obstructive pulmonary disease, cystic fibrosis, asthma, idiopathic pulmonary fibrosis, and critical illness, can disturb the balance of lung microbiota, leading to decreased symbiotic bacteria and increased pathogenic bacteria (Durack et al., 2017; Feigelman et al., 2017; Kim et al., 2017; Dickson et al., 2020; Ramírez-Labrada et al., 2020). These pulmonary diseases can change the immigration, elimination, and growth conditions, which subsequently alter the environment in favor of the growth of certain microbes, ultimately influencing the composition of the lung microbiota. These changes lead to a decrease in the bacterial diversity of the lung microbiota and are associated with the occurrence or progression of lung diseases (Dickson et al., 2016). However, the changing patterns of the lung microbiota are conflicting and inconsistent among different studies. Different specimen types, life styles, drug usage, and other environmental factors, such as diet, smoking, alcohol consumption, and air pollution, affect the structure and composition of the lung microbiota, which would contribute to the inconsistent findings among different studies (Taylor et al., 2019).

Lee et al. found differences in the BALF bacterial communities of Korean patients with LC and those with benign mass-like lesions, with an increased abundance of *Veillonella*, *Megasphaera*, *Atopobium*, and *Selenomonas* in patients with LC. The genera *Veillonella* and *Megasphaera* have the potential to serve as biomarkers for predicting LC (Lee et al., 2016). With protected bronchial specimen brushing samples, Liu et al. found that significant decreases in microbial diversity were observed in Chinese patients with LC compared with healthy controls. The genus *Streptococcus* was significantly more abundant in cancer cases than in controls, while *Staphylococcus* was more abundant in controls (Liu et al., 2018). One of the largest studies on lung microbiota, which included 165 non-malignant and 31 paired tumors, found decreased α diversity in paired tumors, and identified more *Thermus* in tissues from advanced stage (IIIB, IV) patients and more *Legionella* in patients who developed metastases (Yu et al., 2016). Our present study also observed increased bacterial α-diversity and several key functional genera, such as *Sphingobium*, *Marseilla*, *Neisseria*, *Megamonas* and *Fusobacterium*. The interaction network showed stronger positive and negative correlations between these bacteria in the LC group. Several bacteria have been associated with chronic inflammation and an increased LC risk. A significant relationship between *M. tuberculosis* and LC development has been previously reported (Liang et al., 2009). The roles and precise mechanisms of action of these bacteria in lung carcinogenesis have not been fully explored. Recent evidence suggests that dysbiosis of the lung microbiota may modulate the risk of malignancy at multiple levels (Greathouse et al., 2018). One possible mechanism might be that the lung microbiota influenced chronic inflammation by promoting pro-inflammatory factors that stimulate airway epithelial cell proliferation (oncogenes), which ultimately induces cell transformation and initiates tumor formation (Ramírez-Labrada et al., 2020; Zhao et al., 2021). Future studies, both in animal models and patients, are necessary to validate these findings and to gain deeper knowledge of the composition of lung microbiota and their role in LC, before they can be used as cancer biomarkers or included in therapeutic approaches.

PTB is caused by *M tuberculosis*, an intracellular airborne pathogen. A previous study found that dysbiosis of normal lung microbiota is important in the pathogenesis of *M. tuberculosis* (Tarashi et al., 2018). One Chinese study enrolled 32 patients with PTB and 24 healthy individuals and collected BALF samples for pyrosequencing. They found that the lung microbiota in PTB cases was significantly different from that in healthy controls, with *Cupriavidus* being the dominant genus in PTB cases. Two key functional differential genera, *Mycobacterium* and *Porphyromonas*, have been identified as co-factors in lesion formation (Zhou et al., 2015). Hu et al. also identified distinct features of lung microbiota between samples from Chinese patients with and without *M. tuberculosis*, with decreased α-diversity in *M. tuberculosis*-positive patients and separated β-diversity. *M. tuberculosis*-positive patients were enriched with *Anoxybacillus*, whereas *M. tuberculosis*-negative patients were enriched with *Prevotella, Alloprevotella, Veillonella*, and *Gemella* (Hu et al., 2020). The present study also found that *Mycobacterium*, *Selenomonas*, *Leptotrichia*, *Campylobacter*, and *Lactobacillus* were enriched in patients with PTB. Another American study observed a significant reduction in the abundance of *Streptococcus* and *Fusobacterium* genera and increased *Mycobacterium* abundance in BALF samples from patients with PTB (Vázquez-Pérez et al., 2020). One review summarized recent findings on the composition and diversity of lung microbiota among patients with PTB and healthy individuals. Genus-level analysis revealed that *Streptococcus* (35.01%), *Neisseria* (27.1%), *Prevotella* (9.02%) and *Veillonella* (7.8%) were abundant in patients with PTB, whereas some specific bacterial genera, such as *Veillonella*, *Rothia*, *Leuconostoc*, and *Lactobacillus*, were present only in patients with PTB (Eshetie and Van Soolingen, 2019). Interestingly, BALF inflammation was more severe in patients with PTB than in those with LC and CAP, suggesting that dysbiosis of the lung microbiota in PTB may promote lung inflammation. Taken together, these alterations in the lung microbiota and its corresponding host response play a role in the pathophysiological processes of PTB.

Recent data, including the present analysis, suggest that dysbiosis of the lung microbiota likely plays a role in CAP. Mounting evidence has confirmed that CAP results from complex interactions between the host immune response, the respiratory tract microbiota, and CAP-associated pathogens. Lung commensals may contribute to the clinical course of CAP by altering host immune responses or the virulence of potential pathogens in a synergistic or additive manner. Pettigrew et al. observed that certain taxa in sputum microbiota profiles were associated with the severity of CAP in children (Pettigrew et al., 2016). Wootton et al. analyzed sputum samples from adult patients with CAP using pyrosequencing and found that *Veillonella* and *Streptococcus* were the most abundant taxa (Wootton et al., 2019). However, most of the aforementioned studies on CAP examined upper respiratory tract samples, which could not represent the lower respiratory tract microbiota. Our study found significant alterations in the lung microbiota of CAP group compared to the LC and PTB groups. Most of the key functional taxa were decreased and only a few genera, such as *Phenylobacterium*, *Lautropia*, *Parvimonas*, *Deinococcus_Thermus*, and *Deinococcus*, were enriched in the CAP group. A previous study investigated the lung microbiota using BALF samples from Ugandan patients with human immunodeficiency virus and pneumonia using the MiSeq sequencing platform. They identified three distinct community profiles in BALF samples, and the groups differed in metagenomic functional capacity, immune responses in the lower airways, and circulating metabolites (Shenoy et al., 2017). Similar to patients with LC, we also observed low BALF inflammation in patients with CAP. However, research on the lung microbiota is still in its infancy and their role in CAP remains unknown. Dickson et al. proposed ecological models of pneumonia, which posited that its pathogenesis involves a rapid shift from a homeostatic state in the lung microbiome toward a state of dysbiosis characterized by low microbial diversity, high microbial burden, and host inflammation (Dickson et al., 2014). Determining the possible roles and mechanisms of lung microbiota, especially key functional bacteria, would provide novel diagnostic and therapeutic targets for CAP.

However, this study has several limitations. First, healthy lung microbiota were not included in the present comparative study, as sampling BALF from healthy subjects for research purposes was difficult owing to the invasive nature of the process. Lung microbiota were not static, and there was no consensus on the composition of a healthy or normal lung microbiota. Future studies should recruit healthy volunteers to obtain BALF samples for lung microbiota analysis. Second, we only collected the BALF samples for microbiota analysis, while the samples from the upper respiratory tract, such as nasopharyngeal swabs, were not considered. Understanding the similarity and divergence of microbiota across the airways in different respiratory disorders remains an important area for future research. Third, the number of patients with respiratory disorders was relatively small in our study due to the strict inclusion criteria; more patients from different regions of China should be recruited to further validate our results. Finally, our comparative study only reported correlations among the lung microbiota, BALF cytokines, and different respiratory disorders, however, the causal effects of the lung microbiota on these disorders were not explored. We could not determine whether these dysbiotic changes preceded or were a consequence of the inflammatory process in BALF. Future mechanistic studies in animal models should be performed to verify the causal effects of these key functional bacteria in different respiratory disorders.

In summary, our present study compared the overall structure and composition of the pulmonary microbiota among patients with PTB, LC, and CAP using a high-throughput MiSeq sequencing technique for the first time. We found increased bacterial α-diversity and richness in LC group, whereas β-diversity was not significantly different among the three groups. The altered pulmonary microbiota profiles were identified by LEfSe, demonstrating that the CAP-associated pulmonary microbiota were significantly different between the PTB and LC groups. Several key functionally differential genera can be used as potential diagnostic and therapeutic targets for different respiratory disorders. The predicted bacterial functions of the pulmonary microbiota were also significantly different among the three groups. Additionally, different BALF cytokine profiles were observed among the three respiratory disorders, which correlated with the key functional bacteria in the pulmonary microbiota. Thus, the present comparative study provides evidence supporting the associations among altered lung microbiota, BALF inflammation, and different respiratory disorders, which may improve our understanding of the possible roles and mechanisms of the pulmonary microbiota in the progression of various respiratory disorders.

## Data availability statement

The datasets presented in this study can be found in online repositories. The names of the repository/repositories and accession number(s) can be found in the article/**Supplementary Material**.

## Ethics statement

The studies involving human participants were reviewed and approved by the Ethics Committee of Changxing People’s Hospital (Huzhou, Zhejiang, China). The patients/participants provided their written informed consent to participate in this study.

## Author contributions

LZ, ZL, XX, and JC conceived and designed the experiments. XX, JC, YC, FC, HLu, JL, LW, FP, YW, HLi, DC, ZZ, ZX, MF, ZL, and LZ performed the experiments. ZL, XX, and YC analyzed the data. ZL, and XX wrote the paper and edited the manuscript. The final manuscript was read and approved by all authors.

## Funding

This present work was funded by the grants of Zhejiang Basic Public Welfare Research Project (LGF19H010001), the Nutrition and Care of Maternal & Child Research Fund Project of Guangzhou Biostime Institute of Nutrition & Care (2019BINCMCF045), the National Natural Science Foundation of China (81771724, 31700800, 81790631), Key R&D Program of Zhejiang (2022C03060), the Research Project of Jinan Microecological Biomedicine Shandong Laboratory (JNL- 2022033C), the Taishan Scholar Foundation of Shandong Province (tsqn202103119), the National S&T Major Project of China (2018YFC2000500), and the Foundation of China’s State Key Laboratory for Diagnosis and Treatment of Infectious Diseases.

## Acknowledgments

The authors thank all the participants who recruited patients in this study.

## Conflict of interest

The authors declare that the research was conducted in the absence of any commercial or financial relationships that could be construed as a potential conflict of interest.

## Publisher’s note

All claims expressed in this article are solely those of the authors and do not necessarily represent those of their affiliated organizations, or those of the publisher, the editors and the reviewers. Any product that may be evaluated in this article, or claim that may be made by its manufacturer, is not guaranteed or endorsed by the publisher.

## Supplementary material

Supplementary Figure 1 Krona charts showing the taxonomic identification and relative abundance of the most abundant bacterial OTUs recorded among patients with primary pulmonary tuberculosis (PTB), newly diagnosed lung cancer (LC), and community-acquired pneumonia (CAP). These taxa represent the internal core microbiota at the individual level.

Supplementary Figure 2 Taxonomic differences of the lung microbiota among three respiratory diseases. LEfSe identified the most differentially abundant taxa between the two groups. Only the taxa meeting a significant LDA threshold value of > 2 are shown.
