# Supplementary figures and images for: Comparative analysis of the lung microbiota in patients with respiratory infections, tuberculosis, and lung cancer: A preliminary study
Source: Front Cell Infect Microbiol. 2022 Nov 1;12:1024867. doi: 10.3389/fcimb.2022.1024867 (PMC9663837; doi:10.3389/fcimb.2022.1024867)

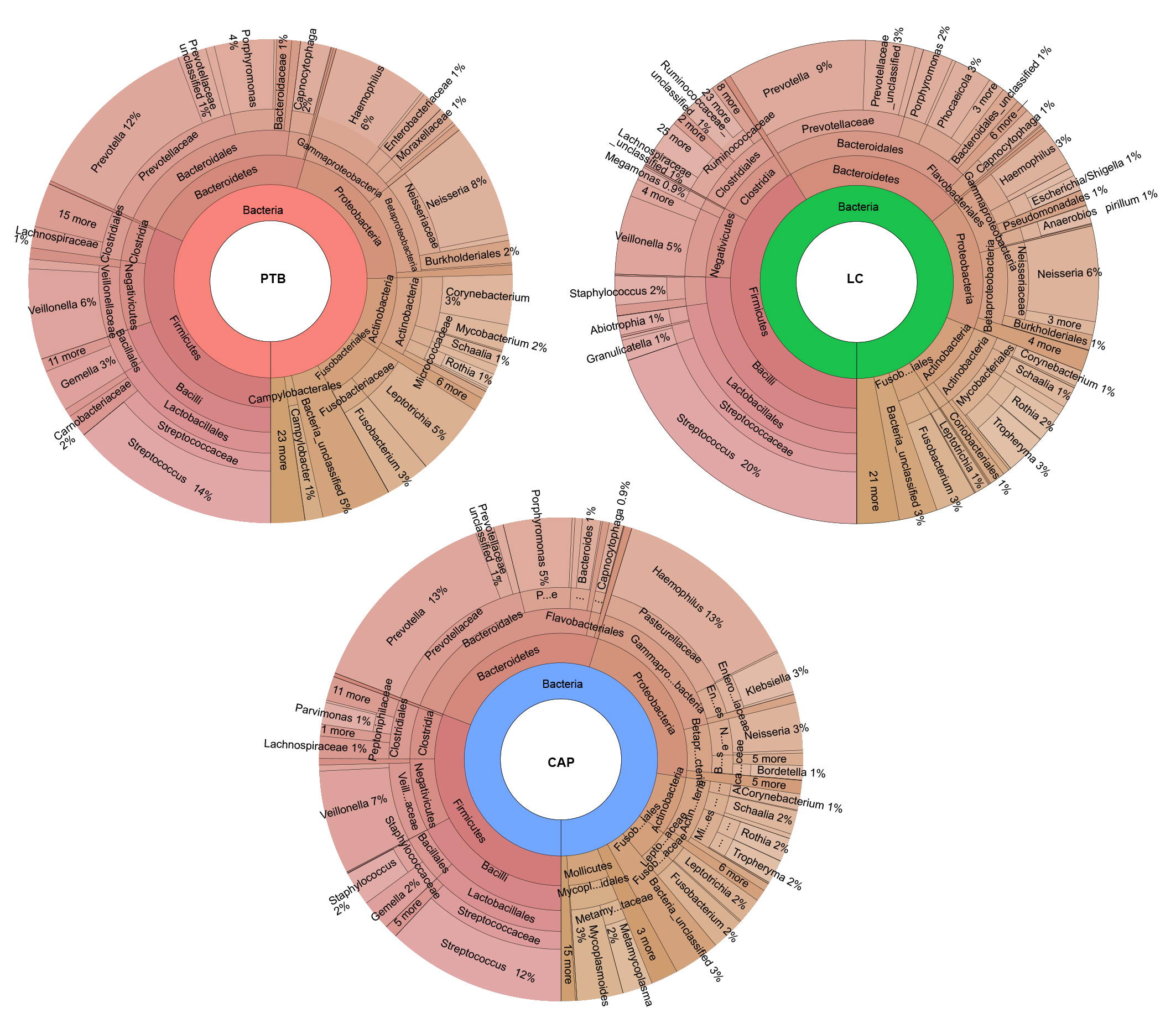

Supplement: Supplementary Figure 1 — Krona charts showing the taxonomic identification and relative abundance of the most abundant bacterial OTUs recorded among patients with primary pulmonary tuberculosis (PTB), newly diagnosed lung cancer (LC), and community-acquired pneumonia (CAP). These taxa represent the internal core microbiota at the individual level. [file Image_1.jpeg]

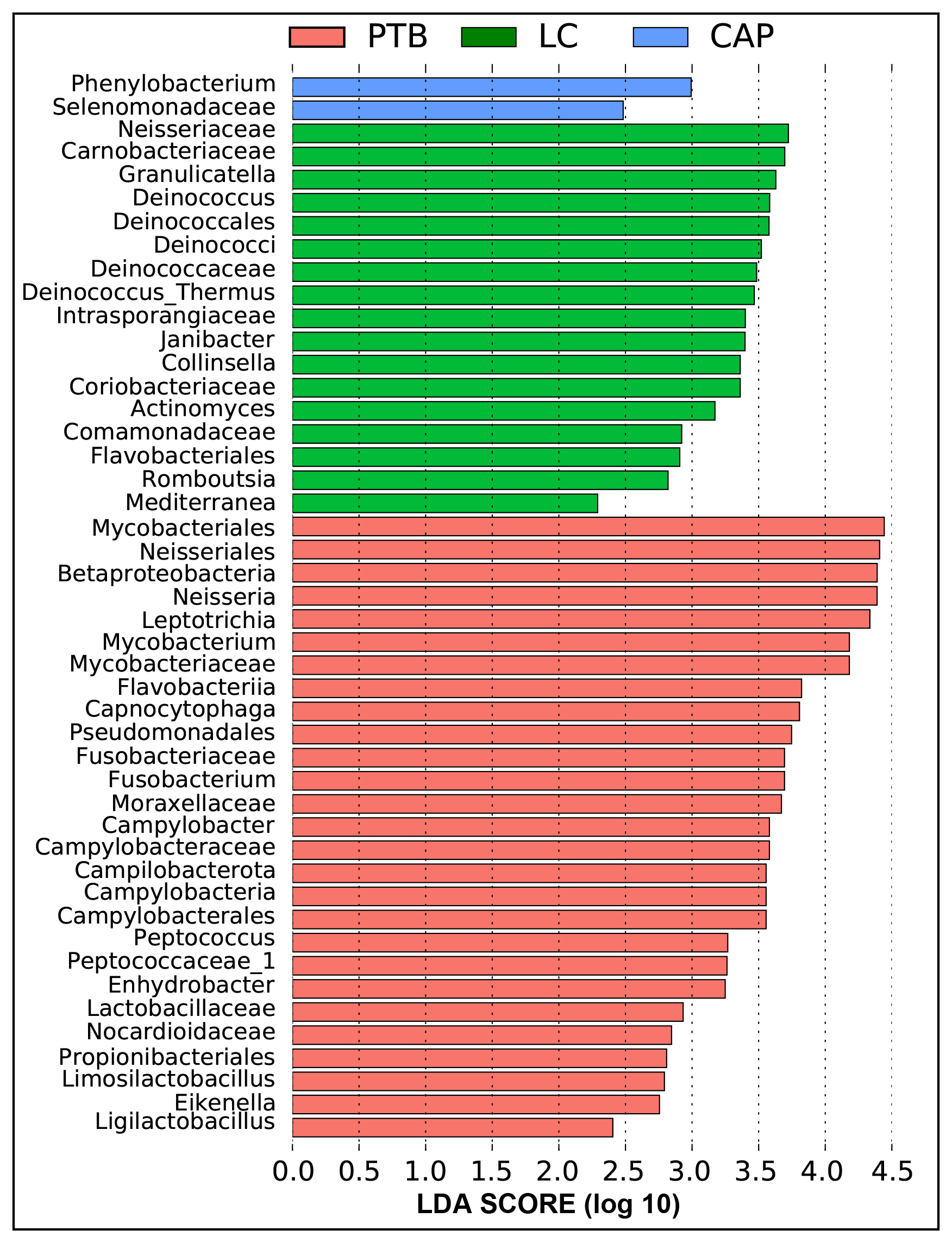

Supplement: Supplementary Figure 2 — Taxonomic differences of the lung microbiota among three respiratory diseases. LEfSe identified the most differentially abundant taxa between the two groups. Only the taxa meeting a significant LDA threshold value of > 2 are shown. [file Image_2.jpeg]
